# Pyridinium tetra­kis­(1,1,1-trifluoro­pentane-2,4-dionato)dysprosate

**DOI:** 10.1107/S1600536811005034

**Published:** 2011-02-16

**Authors:** Yan Wang, Yuekui Wang, Jie Jia, Xiaoli Gao, Xiaoling Su

**Affiliations:** a Department of Chemistry & Chemical Engineering, Luliang University, Lvliang 033000, People’s Republic of China; b Institute of Molecular Science, Key Laboratory of Chemical Biology and Molecular, Engineering of the Education Ministry, Shanxi University, Taiyuan, Shanxi 030006, People’s Republic of China

## Abstract

In the anion of the title compound, (C_5_H_6_N)[Dy(C_5_H_4_F_3_O_2_)_4_], the central metal ion, Dy^3+^, is coordinated by four bidentate 1,1,1-trifluoro­pentane-2,4-dionate (TAA) ligands, forming an approximate square-anti­prismatic configuration. The pyridin­ium cation is connected to the complex ion by an N—H⋯O hydrogen bond and electrostatic inter­actions in the crystal. There are two kinds of disorder in the structure, one involving rotational disorder of a CF_3_ group [occupancy ratio 0.560 (15):0.440 (15)] and the other involving an exchange between a CF_3_ group and CH_3_ group within a given bidentate ligand (occupancy ratio 0.64:0.36).

## Related literature

For applications of rare earth–β-diketone complexes, see: Chu & Elgavish (1995 ▶); Tsukube & Shinoda (2002 ▶); Iwamuro *et al.* (1997 ▶). For related structures, see: Ma *et al.* (2000 ▶); Tian *et al.* (2009 ▶).
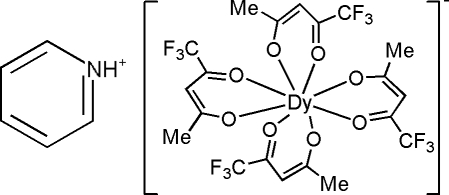


## Experimental

### 

#### Crystal data

(C_5_H_6_N)[Dy(C_5_H_4_F_3_O_2_)_4_]
*M* *_r_* = 854.94
Monoclinic, 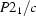

*a* = 10.619 (4) Å
*b* = 19.799 (7) Å
*c* = 15.715 (6) Å
β = 103.116 (6)°
*V* = 3217.8 (19) Å^3^
*Z* = 4
Mo *K*α radiation
μ = 2.44 mm^−1^
*T* = 298 K
0.22 × 0.22 × 0.06 mm

#### Data collection

Bruker SMART 1K CCD area detector diffractometer
Absorption correction: multi-scan (*SADABS*; Sheldrick, 2000 ▶) *T*_min_ = 0.616, *T*_max_ = 0.868
14449 measured reflections
5670 independent reflections
4126 reflections with *I* > 2σ(*I*)
*R*_int_ = 0.039

#### Refinement

*R*[*F* ^2^ > 2σ(*F* ^2^)] = 0.050
*wR*(*F* ^2^) = 0.121
*S* = 1.02
5670 reflections
483 parameters
78 restraints
H-atom parameters constrained
Δρ_max_ = 0.95 e Å^−3^
Δρ_min_ = −0.45 e Å^−3^

### 

Data collection: *SMART* (Bruker, 2000 ▶); cell refinement: *SAINT* (Bruker, 2000 ▶); data reduction: *SAINT*; program(s) used to solve structure: *SHELXS97* (Sheldrick, 2008 ▶); program(s) used to refine structure: *SHELXL97* (Sheldrick, 2008 ▶); molecular graphics: *ORTEP-3* (Farrugia, 1997 ▶); software used to prepare material for publication: *SHELXTL/PC* (Sheldrick, 2008 ▶).

## Supplementary Material

Crystal structure: contains datablocks I, global. DOI: 10.1107/S1600536811005034/om2404sup1.cif

Structure factors: contains datablocks I. DOI: 10.1107/S1600536811005034/om2404Isup2.hkl

Additional supplementary materials:  crystallographic information; 3D view; checkCIF report

## Figures and Tables

**Table 1:** Selected bond lengths (Å)

Dy1—O2	2.305 (5)
Dy1—O6	2.315 (5)
Dy1—O3	2.326 (5)
Dy1—O4	2.342 (5)
Dy1—O8	2.349 (4)
Dy1—O7	2.356 (5)
Dy1—O5	2.390 (5)
Dy1—O1	2.423 (5)

**Table 2:** Hydrogen-bond geometry (Å, °)

*D*—H⋯*A*	*D*—H	H⋯*A*	*D*⋯*A*	*D*—H⋯*A*
N1—H1⋯O5	0.86	2.10	2.947 (8)	167

**Fig. 1.**
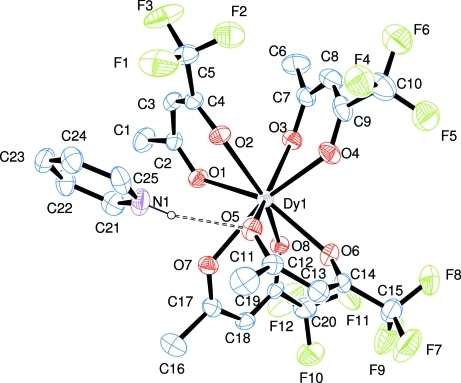
A view of the structure of the complex with displacement ellipsoids drawn at the 30% probability level. H atoms without H-bond (dotting line) and minor disorder components were omitted for clarity.

## supplementary crystallographic information

### Comment

Rare earth-β-diketone complexes have attracted considerable attention in the past decades owing to their important applications as laser (Iwamuro *et al.*, 1997), fluorescent probe (Tsukube & Shinoda, 2002) and NMR reagents (Chu & Elgavish, 1995). As part of our interest in this field, we have been engaged in a major effort directed toward the development of syntheses of new lanthanide-β-diketon complexes.

The structure of the title Dy^3+^ complex is shown in Fig. 1. It contains an eight-coordinate dysprosium ion bonded to four TAA anions with bidentate chelation, forming the [Dy(TAA)_4_]^-^ anions. These are connected to pyridinium cations by a N—H···O hydrogen bond. The co-ordination polyhedron may be described as an approximate square antiprism and the two sets of four O atoms (O2, O4, O5, O7) and (O1, O3, O6, O8) form the twisted upper and lower sides respectively. The Dy—O bond lengths are in the range of 2.305 (5)–2.423 (5) Å, average 2.351 (2) Å, which is consistent with other work in literature (Ma *et al.*, 2000; Tian *et al.*, 2009). The average angle of O—Dy—O is 100.25° and the average dihydral angle (C—O—Dy—O) is -10.49°.

### Experimental

A mixture of Dy_2_O_3_ (0.186 g) and concentrated hydrochloric (5 mL) was heated and distilled to slight dryness, yielding a crystalline precipitate (DyCl_3_). Then, the DyCl_3_ solid was redissolved in 5 mL absolute ethanol, and heated with 10 mL of absolute ethanol solution containing HTAA (0.50 mL) and pyridine (0.32 mL) at about 363 K. The reaction mixture was maintained at ambient temperature for one month until yellow crystals formed.

### Refinement

All F atoms were found to be disordered. There is disorder of the two different types: 1) disorder due to rotational disorder of the CF_3_ group bonded to a single carbon. F4, F5, and F6 atoms was split into to two sets of positions using restraints on their anisotropic displacement parameters. The major and minor disorder components had refined occupancies of 0.56 (2) and 0.44 (2), respectively; 2) disorder due to exchange of CH_3_ and CF_3_ groups on the same ligand. Namely, F10, F11, and F12 as well as related H atoms were modelled over two sets of positions using restraint on their anisotropic displacement parameters. The major and minor disorder components had refined occupancies of 0.64 (1) and 0.36 (1), respectively. In the final refinement, the occupancies of these disordered atoms were fixed to aid convergence. Atoms F11B, F4, F5, F6, F4B, F5B, and F6B were refined anisotropically using 42 restraints (ISOR) and the geometrical parameters of CF_3_ group were refined using 36 restraints (*DFIX* and DANG) because of the unacceptable parameters of their ellipsoids and distances between atoms. H atoms attached to C and N were placed in geometrically idealized positions with C*sp*^2^—H = 0.93, C*sp*^3^—H = 0.96, N*sp*^2^—H = 0.86 Å, and constrained to ride on their carrier atoms, with Uĩso~(H) = 1.2Ũeq~(C & N) and Uĩso~(H) = 1.5Ũeq~(C).

### Crystal data

**Table:**

(C_5_H_6_N)[Dy(C_5_H_4_F_3_O_2_)_4_]	*F*(000) = 1668
*M**_r_* = 854.94	*D*_x_ = 1.765 Mg m^−^^3^
Monoclinic, *P*2_1_/*c*	Mo *K*α radiation, λ = 0.71073 Å
Hall symbol: -P 2ybc	Cell parameters from 3207 reflections
*a* = 10.619 (4) Å	θ = 2.2–21.0°
*b* = 19.799 (7) Å	µ = 2.44 mm^−^^1^
*c* = 15.715 (6) Å	*T* = 298 K
β = 103.116 (6)°	Plate, colorless
*V* = 3217.8 (19) Å^3^	0.22 × 0.22 × 0.06 mm
*Z* = 4	

### Data collection

**Table:**

Bruker SMART 1K CCD area detector diffractometer	5670 independent reflections
Radiation source: fine-focus sealed tube	4126 reflections with *I* > 2σ(*I*)
graphite	*R*_int_ = 0.039
ω scans	θ_max_ = 25.0°, θ_min_ = 1.7°
Absorption correction: multi-scan (*SADABS*; Sheldrick, 2000)	*h* = −12→12
*T*_min_ = 0.616, *T*_max_ = 0.868	*k* = −19→23
14449 measured reflections	*l* = −18→15

### Refinement

**Table:**

Refinement on *F*^2^	Primary atom site location: structure-invariant direct methods
Least-squares matrix: full	Secondary atom site location: difference Fourier map
*R*[*F*^2^ > 2σ(*F*^2^)] = 0.050	Hydrogen site location: inferred from neighbouring sites
*wR*(*F*^2^) = 0.121	H-atom parameters constrained
*S* = 1.02	*w* = 1/[σ^2^(*F*_o_^2^) + (0.0621*P*)^2^] where *P* = (*F*_o_^2^ + 2*F*_c_^2^)/3
5670 reflections	(Δ/σ)_max_ = 0.001
483 parameters	Δρ_max_ = 0.95 e Å^−^^3^
78 restraints	Δρ_min_ = −0.45 e Å^−^^3^

### Special details

**Table:**

Geometry. All e.s.d.'s (except the e.s.d. in the dihedral angle between two l.s. planes) are estimated using the full covariance matrix. The cell e.s.d.'s are taken into account individually in the estimation of e.s.d.'s in distances, angles and torsion angles; correlations between e.s.d.'s in cell parameters are only used when they are defined by crystal symmetry. An approximate (isotropic) treatment of cell e.s.d.'s is used for estimating e.s.d.'s involving l.s. planes.
Refinement. Refinement of *F*^2^ against ALL reflections. The weighted *R*-factor *wR* and goodness of fit *S* are based on *F*^2^, conventional *R*-factors *R* are based on *F*, with *F* set to zero for negative *F*^2^. The threshold expression of *F*^2^ > σ(*F*^2^) is used only for calculating *R*-factors(gt) *etc*. and is not relevant to the choice of reflections for refinement. *R*-factors based on *F*^2^ are statistically about twice as large as those based on *F*, and *R*- factors based on ALL data will be even larger.

### Fractional atomic coordinates and isotropic or equivalent isotropic displacement parameters (Å^2^)

**Table:**

	*x*	*y*	*z*	*U*_iso_*/*U*_eq_	Occ. (<1)
Dy1	0.54349 (3)	0.155070 (15)	0.274306 (19)	0.05328 (14)	
O1	0.4836 (5)	0.2430 (2)	0.3649 (3)	0.0698 (13)	
O2	0.3848 (5)	0.2201 (2)	0.1874 (3)	0.0664 (13)	
C1	0.3730 (10)	0.3186 (5)	0.4363 (6)	0.105 (3)	
H1A	0.4141	0.2953	0.4889	0.157*	
H1B	0.2835	0.3259	0.4360	0.157*	
H1C	0.4147	0.3614	0.4339	0.157*	
C2	0.3834 (8)	0.2772 (4)	0.3590 (5)	0.0656 (19)	
C3	0.2847 (8)	0.2808 (4)	0.2828 (5)	0.069 (2)	
H3	0.2086	0.3029	0.2860	0.083*	
C4	0.2930 (7)	0.2543 (4)	0.2051 (5)	0.0633 (19)	
C5	0.1851 (9)	0.2668 (5)	0.1261 (6)	0.088 (3)	
F1	0.2181 (6)	0.3041 (4)	0.0687 (4)	0.170 (3)	
F2	0.1395 (7)	0.2116 (4)	0.0866 (5)	0.165 (3)	
F3	0.0817 (7)	0.2960 (4)	0.1425 (4)	0.170 (3)	
O3	0.3793 (5)	0.1120 (2)	0.3346 (3)	0.0691 (13)	
O4	0.4291 (6)	0.0745 (3)	0.1777 (3)	0.0813 (15)	
C6	0.1664 (9)	0.1026 (6)	0.3557 (6)	0.116 (3)	
H6A	0.1652	0.0631	0.3908	0.173*	
H6B	0.0825	0.1092	0.3180	0.173*	
H6C	0.1886	0.1413	0.3930	0.173*	
C7	0.2647 (9)	0.0938 (4)	0.3014 (6)	0.076 (2)	
C8	0.2294 (10)	0.0660 (5)	0.2176 (7)	0.097 (3)	
H8	0.1438	0.0530	0.1968	0.116*	
C9	0.3145 (12)	0.0569 (4)	0.1642 (5)	0.090 (3)	
C10	0.2692 (12)	0.0137 (6)	0.0779 (8)	0.129 (4)	
F4	0.2652 (17)	0.0495 (7)	0.0147 (8)	0.139 (5)	0.560 (15)
F5	0.3418 (14)	−0.0434 (6)	0.0845 (8)	0.138 (5)	0.560 (15)
F6	0.1486 (13)	−0.0078 (8)	0.0698 (9)	0.152 (6)	0.560 (15)
F4B	0.237 (2)	−0.0437 (7)	0.0915 (10)	0.136 (6)	0.440 (15)
F5B	0.158 (2)	0.0463 (12)	0.0364 (15)	0.201 (10)	0.440 (15)
F6B	0.3444 (16)	0.0219 (8)	0.0223 (10)	0.123 (6)	0.440 (15)
O5	0.6258 (5)	0.1812 (2)	0.1486 (3)	0.0685 (13)	
O6	0.6880 (5)	0.0700 (2)	0.2623 (3)	0.0687 (13)	
C11	0.7528 (9)	0.1973 (5)	0.0427 (5)	0.103 (3)	
H11A	0.6789	0.2041	−0.0045	0.155*	
H11B	0.8178	0.1727	0.0219	0.155*	
H11C	0.7869	0.2402	0.0652	0.155*	
C12	0.7130 (8)	0.1571 (4)	0.1151 (5)	0.0664 (19)	
C13	0.7781 (8)	0.0965 (4)	0.1434 (5)	0.075 (2)	
H13	0.8392	0.0817	0.1135	0.089*	
C14	0.7591 (7)	0.0577 (3)	0.2112 (5)	0.0641 (19)	
C15	0.8293 (10)	−0.0080 (5)	0.2295 (7)	0.093 (3)	
F7	0.9130 (8)	−0.0209 (3)	0.1817 (6)	0.170 (3)	
F8	0.7516 (7)	−0.0582 (3)	0.2151 (6)	0.185 (4)	
F9	0.8939 (9)	−0.0126 (4)	0.3073 (5)	0.194 (4)	
O7	0.7171 (5)	0.2319 (2)	0.3091 (3)	0.0692 (13)	
O8	0.6620 (5)	0.1262 (2)	0.4147 (3)	0.0624 (12)	
C16	0.9245 (11)	0.2782 (6)	0.3435 (7)	0.104 (3)	
H16A	0.9699	0.2688	0.2962	0.104*	0.64
H16B	1.0012	0.2854	0.3963	0.104*	0.64
H16C	0.8937	0.3261	0.3317	0.104*	0.64
F10B	1.0364 (15)	0.2718 (10)	0.3858 (12)	0.157 (8)	0.36
F11B	0.924 (2)	0.2781 (10)	0.2596 (11)	0.160 (7)	0.36
F12B	0.879 (2)	0.3390 (8)	0.3519 (18)	0.193 (12)	0.36
C17	0.8278 (8)	0.2252 (4)	0.3569 (5)	0.068 (2)	
C18	0.8665 (8)	0.1759 (4)	0.4206 (5)	0.070 (2)	
H18	0.9536	0.1735	0.4483	0.084*	
C19	0.7838 (7)	0.1309 (3)	0.4447 (4)	0.0589 (18)	
C20	0.8358 (10)	0.0834 (5)	0.5191 (6)	0.099 (3)	
H20A	0.7625	0.0425	0.5206	0.099*	0.36
H20B	0.8509	0.0975	0.5761	0.099*	0.36
H20C	0.9073	0.0496	0.5146	0.099*	0.36
F10	0.9621 (9)	0.0751 (5)	0.5361 (6)	0.141 (3)	0.64
F11	0.7907 (13)	0.0241 (5)	0.5025 (9)	0.243 (9)	0.64
F12	0.8159 (13)	0.1056 (7)	0.5915 (6)	0.194 (6)	0.64
N1	0.5608 (8)	0.3261 (3)	0.1375 (6)	0.096 (2)	
H1	0.5924	0.2859	0.1424	0.115*	
C21	0.5610 (9)	0.3615 (5)	0.2096 (6)	0.089 (3)	
H21	0.5963	0.3435	0.2645	0.106*	
C22	0.5089 (10)	0.4241 (4)	0.2015 (6)	0.088 (3)	
H22	0.5072	0.4495	0.2510	0.105*	
C23	0.4599 (9)	0.4493 (4)	0.1219 (6)	0.089 (3)	
H23	0.4224	0.4920	0.1162	0.106*	
C24	0.4647 (9)	0.4130 (4)	0.0494 (6)	0.088 (3)	
H24	0.4341	0.4316	−0.0057	0.106*	
C25	0.5124 (10)	0.3516 (4)	0.0571 (7)	0.089 (3)	
H25	0.5129	0.3259	0.0076	0.107*	

### Atomic displacement parameters (Å^2^)

**Table:**

	*U*^11^	*U*^22^	*U*^33^	*U*^12^	*U*^13^	*U*^23^
Dy1	0.0585 (2)	0.0521 (2)	0.0466 (2)	0.00072 (16)	0.00630 (14)	0.00471 (15)
O1	0.067 (3)	0.074 (3)	0.062 (3)	0.006 (3)	0.002 (2)	−0.007 (2)
O2	0.062 (3)	0.078 (3)	0.054 (3)	0.011 (3)	0.004 (2)	0.009 (2)
C1	0.123 (9)	0.100 (6)	0.092 (7)	0.029 (6)	0.027 (6)	−0.018 (5)
C2	0.068 (5)	0.054 (4)	0.075 (5)	0.003 (4)	0.017 (4)	0.007 (4)
C3	0.068 (5)	0.063 (5)	0.076 (5)	0.015 (4)	0.013 (4)	0.009 (4)
C4	0.058 (5)	0.062 (4)	0.063 (5)	0.004 (4)	−0.001 (4)	0.020 (4)
C5	0.072 (6)	0.108 (7)	0.077 (6)	0.010 (6)	0.000 (5)	0.014 (6)
F1	0.125 (5)	0.248 (8)	0.120 (5)	−0.011 (5)	−0.008 (4)	0.111 (6)
F2	0.137 (6)	0.161 (6)	0.148 (6)	−0.001 (5)	−0.067 (4)	−0.004 (5)
F3	0.106 (5)	0.275 (9)	0.111 (5)	0.088 (6)	−0.010 (4)	0.003 (5)
O3	0.071 (4)	0.071 (3)	0.063 (3)	−0.009 (3)	0.009 (3)	0.010 (3)
O4	0.084 (4)	0.085 (4)	0.071 (3)	−0.020 (3)	0.008 (3)	−0.026 (3)
C6	0.078 (7)	0.159 (10)	0.122 (8)	−0.001 (7)	0.049 (6)	0.015 (7)
C7	0.075 (6)	0.065 (5)	0.083 (6)	−0.013 (4)	0.007 (5)	0.014 (4)
C8	0.081 (7)	0.105 (7)	0.096 (7)	−0.021 (5)	0.002 (6)	0.004 (6)
C9	0.123 (9)	0.069 (5)	0.063 (5)	−0.020 (6)	−0.008 (5)	−0.006 (4)
C10	0.130 (11)	0.126 (11)	0.122 (10)	−0.037 (9)	0.008 (8)	0.026 (8)
F4	0.147 (7)	0.143 (7)	0.122 (6)	−0.008 (5)	0.021 (5)	−0.001 (4)
F5	0.145 (7)	0.131 (7)	0.130 (6)	0.002 (4)	0.015 (4)	−0.025 (4)
F6	0.148 (7)	0.151 (7)	0.151 (7)	−0.022 (5)	0.020 (5)	−0.006 (5)
F4B	0.143 (8)	0.128 (7)	0.137 (7)	−0.008 (5)	0.030 (5)	−0.011 (5)
F5B	0.201 (11)	0.203 (11)	0.194 (11)	−0.002 (5)	0.033 (5)	−0.008 (5)
F6B	0.127 (7)	0.125 (7)	0.115 (7)	−0.006 (5)	0.025 (5)	−0.012 (5)
O5	0.083 (4)	0.067 (3)	0.058 (3)	0.006 (3)	0.021 (3)	0.011 (2)
O6	0.085 (4)	0.061 (3)	0.064 (3)	0.015 (3)	0.024 (3)	0.005 (2)
C11	0.118 (8)	0.129 (8)	0.078 (6)	0.001 (7)	0.053 (6)	0.021 (6)
C12	0.064 (5)	0.077 (5)	0.057 (4)	−0.002 (4)	0.013 (4)	−0.010 (4)
C13	0.078 (6)	0.081 (6)	0.071 (5)	0.001 (5)	0.030 (4)	−0.014 (4)
C14	0.065 (5)	0.051 (4)	0.070 (5)	−0.005 (4)	0.002 (4)	−0.018 (4)
C15	0.099 (8)	0.077 (6)	0.108 (8)	0.014 (6)	0.034 (6)	0.001 (6)
F7	0.184 (7)	0.125 (5)	0.231 (8)	0.067 (5)	0.112 (7)	0.020 (5)
F8	0.154 (7)	0.062 (3)	0.348 (12)	−0.006 (4)	0.074 (7)	−0.012 (5)
F9	0.267 (10)	0.153 (6)	0.129 (6)	0.118 (7)	−0.024 (6)	0.014 (5)
O7	0.061 (3)	0.072 (3)	0.071 (3)	−0.005 (3)	0.006 (3)	0.007 (3)
O8	0.063 (3)	0.067 (3)	0.053 (3)	0.004 (3)	0.005 (2)	0.012 (2)
C16	0.091 (8)	0.123 (9)	0.095 (7)	−0.029 (7)	0.015 (6)	0.000 (6)
F10B	0.084 (13)	0.21 (2)	0.160 (16)	−0.055 (13)	−0.010 (11)	0.055 (14)
F11B	0.158 (8)	0.168 (8)	0.157 (8)	−0.016 (5)	0.043 (5)	0.003 (5)
F12B	0.15 (2)	0.110 (14)	0.35 (4)	−0.061 (13)	0.14 (2)	−0.024 (16)
C17	0.067 (5)	0.074 (5)	0.064 (5)	−0.008 (4)	0.019 (4)	−0.016 (4)
C18	0.056 (5)	0.076 (5)	0.069 (5)	0.008 (4)	−0.002 (4)	−0.005 (4)
C19	0.060 (5)	0.058 (4)	0.050 (4)	0.017 (4)	−0.004 (3)	−0.006 (3)
C20	0.086 (7)	0.105 (8)	0.085 (7)	0.005 (6)	−0.025 (5)	0.028 (6)
F10	0.107 (8)	0.146 (8)	0.145 (8)	0.029 (6)	−0.020 (6)	0.041 (6)
F11	0.243 (14)	0.124 (8)	0.253 (15)	−0.077 (9)	−0.170 (12)	0.118 (9)
F12	0.239 (14)	0.273 (15)	0.079 (6)	0.122 (12)	0.052 (8)	0.063 (8)
N1	0.114 (7)	0.059 (4)	0.134 (7)	0.011 (4)	0.069 (6)	0.016 (5)
C21	0.098 (7)	0.094 (7)	0.073 (6)	−0.007 (5)	0.017 (5)	0.020 (5)
C22	0.126 (8)	0.065 (5)	0.074 (6)	−0.003 (5)	0.024 (5)	−0.011 (5)
C23	0.098 (7)	0.064 (5)	0.105 (7)	0.017 (5)	0.024 (6)	−0.002 (5)
C24	0.109 (7)	0.080 (6)	0.072 (5)	−0.008 (5)	0.010 (5)	0.017 (5)
C25	0.116 (8)	0.067 (6)	0.100 (7)	−0.023 (5)	0.058 (6)	−0.016 (5)

### Geometric parameters (Å, °)

**Table:**

Dy1—O2	2.305 (5)	C13—H13	0.9300
Dy1—O6	2.315 (5)	C14—C15	1.494 (11)
Dy1—O3	2.326 (5)	C15—F9	1.263 (10)
Dy1—O4	2.342 (5)	C15—F8	1.278 (10)
Dy1—O8	2.349 (4)	C15—F7	1.313 (9)
Dy1—O7	2.356 (5)	O7—C17	1.250 (9)
Dy1—O5	2.390 (5)	O8—C19	1.275 (8)
Dy1—O1	2.423 (5)	C16—F10B	1.230 (14)
O1—C2	1.247 (8)	C16—F12B	1.314 (14)
O2—C4	1.269 (8)	C16—F11B	1.317 (14)
C1—C2	1.492 (10)	C16—C17	1.517 (12)
C1—H1A	0.9600	C16—H16A	0.9912
C1—H1B	0.9600	C16—H16B	1.0321
C1—H1C	0.9600	C16—H16C	1.0056
C2—C3	1.403 (10)	F10B—H16A	1.4259
C3—C4	1.350 (10)	F10B—H16B	0.5180
C3—H3	0.9300	F11B—H16A	0.6894
C4—C5	1.507 (11)	F12B—H16C	0.4623
C5—F1	1.275 (9)	C17—C18	1.392 (10)
C5—F2	1.295 (9)	C18—C19	1.363 (10)
C5—F3	1.317 (9)	C18—H18	0.9300
O3—C7	1.264 (8)	C19—C20	1.505 (10)
O4—C9	1.238 (11)	C20—F11	1.272 (11)
C6—C7	1.501 (11)	C20—F12	1.281 (11)
C6—H6A	0.9600	C20—F10	1.317 (10)
C6—H6B	0.9600	C20—H20A	1.1271
C6—H6C	0.9600	C20—H20B	0.9180
C7—C8	1.397 (12)	C20—H20C	1.0263
C8—C9	1.378 (13)	F10—H20C	0.7857
C8—H8	0.9300	F11—H20A	0.5838
C9—C10	1.582 (14)	F11—H20C	1.3102
C10—F4	1.213 (12)	F12—H20B	0.5114
C10—F4B	1.220 (14)	N1—C21	1.333 (11)
C10—F6B	1.321 (14)	N1—C25	1.350 (12)
C10—F6	1.328 (13)	N1—H1	0.8600
C10—F5	1.359 (13)	C21—C22	1.350 (11)
C10—F5B	1.372 (16)	C21—H21	0.9300
O5—C12	1.258 (8)	C22—C23	1.338 (11)
O6—C14	1.245 (8)	C22—H22	0.9300
C11—C12	1.525 (10)	C23—C24	1.358 (11)
C11—H11A	0.9600	C23—H23	0.9300
C11—H11B	0.9600	C24—C25	1.312 (11)
C11—H11C	0.9600	C24—H24	0.9300
C12—C13	1.405 (10)	C25—H25	0.9300
C13—C14	1.365 (10)		
			
O2—Dy1—O6	140.08 (17)	F6—C10—C9	110.8 (11)
O2—Dy1—O3	86.05 (17)	F5—C10—C9	109.1 (10)
O6—Dy1—O3	109.78 (17)	F5B—C10—C9	102.3 (13)
O2—Dy1—O4	78.12 (19)	C12—O5—Dy1	134.9 (5)
O6—Dy1—O4	73.0 (2)	C14—O6—Dy1	134.8 (5)
O3—Dy1—O4	71.84 (18)	C12—C11—H11A	109.5
O2—Dy1—O8	148.85 (17)	C12—C11—H11B	109.5
O6—Dy1—O8	71.07 (16)	H11A—C11—H11B	109.5
O3—Dy1—O8	79.62 (17)	C12—C11—H11C	109.5
O4—Dy1—O8	122.14 (17)	H11A—C11—H11C	109.5
O2—Dy1—O7	101.87 (18)	H11B—C11—H11C	109.5
O6—Dy1—O7	89.42 (18)	O5—C12—C13	123.2 (7)
O3—Dy1—O7	139.54 (17)	O5—C12—C11	118.0 (7)
O4—Dy1—O7	148.56 (18)	C13—C12—C11	118.7 (7)
O8—Dy1—O7	73.40 (16)	C14—C13—C12	125.3 (7)
O2—Dy1—O5	75.24 (17)	C14—C13—H13	117.4
O6—Dy1—O5	73.54 (16)	C12—C13—H13	117.4
O3—Dy1—O5	149.75 (18)	O6—C14—C13	127.8 (7)
O4—Dy1—O5	81.05 (18)	O6—C14—C15	113.3 (7)
O8—Dy1—O5	127.59 (17)	C13—C14—C15	119.0 (8)
O7—Dy1—O5	68.84 (17)	F9—C15—F8	107.6 (10)
O2—Dy1—O1	72.12 (16)	F9—C15—F7	104.5 (9)
O6—Dy1—O1	146.89 (17)	F8—C15—F7	103.9 (8)
O3—Dy1—O1	72.74 (17)	F9—C15—C14	112.7 (8)
O4—Dy1—O1	134.69 (19)	F8—C15—C14	111.7 (8)
O8—Dy1—O1	77.24 (16)	F7—C15—C14	115.7 (8)
O7—Dy1—O1	72.29 (17)	C17—O7—Dy1	130.6 (5)
O5—Dy1—O1	121.60 (16)	C19—O8—Dy1	127.7 (4)
C2—O1—Dy1	132.8 (5)	F10B—C16—F12B	112.0 (17)
C4—O2—Dy1	131.5 (4)	F10B—C16—F11B	108.9 (15)
C2—C1—H1A	109.5	F12B—C16—F11B	100.6 (16)
C2—C1—H1B	109.5	F10B—C16—C17	117.0 (12)
H1A—C1—H1B	109.5	F12B—C16—C17	110.0 (11)
C2—C1—H1C	109.5	F11B—C16—C17	106.8 (12)
H1A—C1—H1C	109.5	C17—C16—H16A	115.7
H1B—C1—H1C	109.5	C17—C16—H16B	114.7
O1—C2—C3	123.5 (7)	H16A—C16—H16B	101.5
O1—C2—C1	117.3 (7)	C17—C16—H16C	118.4
C3—C2—C1	119.2 (8)	H16A—C16—H16C	103.4
C4—C3—C2	124.2 (7)	H16B—C16—H16C	100.6
C4—C3—H3	117.9	H16A—F10B—H16B	96.0
C2—C3—H3	117.9	C16—F11B—H16A	47.4
O2—C4—C3	128.1 (7)	O7—C17—C18	126.1 (7)
O2—C4—C5	112.7 (7)	O7—C17—C16	114.7 (8)
C3—C4—C5	119.2 (7)	C18—C17—C16	119.1 (8)
F1—C5—F2	106.4 (9)	C19—C18—C17	123.6 (7)
F1—C5—F3	105.0 (8)	C19—C18—H18	118.2
F2—C5—F3	103.1 (9)	C17—C18—H18	118.2
F1—C5—C4	113.5 (8)	O8—C19—C18	127.5 (6)
F2—C5—C4	112.8 (7)	O8—C19—C20	113.8 (7)
F3—C5—C4	115.0 (8)	C18—C19—C20	118.6 (7)
C7—O3—Dy1	132.6 (5)	F11—C20—F12	111.6 (13)
C9—O4—Dy1	130.5 (6)	F11—C20—F10	104.2 (11)
C7—C6—H6A	109.5	F12—C20—F10	103.2 (9)
C7—C6—H6B	109.5	F11—C20—C19	111.3 (8)
H6A—C6—H6B	109.5	F12—C20—C19	111.8 (9)
C7—C6—H6C	109.5	F10—C20—C19	114.4 (9)
H6A—C6—H6C	109.5	C19—C20—H20A	109.4
H6B—C6—H6C	109.5	C19—C20—H20B	121.4
O3—C7—C8	121.9 (8)	H20A—C20—H20B	99.6
O3—C7—C6	117.5 (8)	C19—C20—H20C	120.1
C8—C7—C6	120.6 (9)	H20A—C20—H20C	93.3
C9—C8—C7	123.6 (9)	H20B—C20—H20C	107.3
C9—C8—H8	118.2	C20—F10—H20C	51.2
C7—C8—H8	118.2	C20—F11—H20A	62.4
O4—C9—C8	127.8 (8)	C20—F11—H20C	46.8
O4—C9—C10	113.3 (9)	H20A—F11—H20C	105.2
C8—C9—C10	118.7 (10)	C21—N1—C25	121.7 (7)
F4—C10—F4B	136.8 (15)	C21—N1—H1	119.2
F4—C10—F6B	45.6 (9)	C25—N1—H1	119.2
F4B—C10—F6B	117.9 (15)	N1—C21—C22	118.8 (8)
F4—C10—F6	104.8 (13)	N1—C21—H21	120.6
F4B—C10—F6	54.0 (10)	C22—C21—H21	120.6
F6B—C10—F6	133.7 (13)	C23—C22—C21	119.7 (8)
F4—C10—F5	117.5 (14)	C23—C22—H22	120.1
F4B—C10—F5	52.5 (10)	C21—C22—H22	120.1
F6B—C10—F5	74.6 (12)	C22—C23—C24	120.3 (8)
F6—C10—F5	105.0 (12)	C22—C23—H23	119.8
F4—C10—F5B	57.9 (11)	C24—C23—H23	119.8
F4B—C10—F5B	106.0 (15)	C25—C24—C23	120.0 (9)
F6B—C10—F5B	102.1 (14)	C25—C24—H24	120.0
F6—C10—F5B	53.2 (11)	C23—C24—H24	120.0
F5—C10—F5B	147.1 (15)	C24—C25—N1	119.4 (8)
F4—C10—C9	109.5 (10)	C24—C25—H25	120.3
F4B—C10—C9	113.3 (11)	N1—C25—H25	120.3
F6B—C10—C9	112.8 (11)		
			
O2—Dy1—O1—C2	24.6 (6)	O7—Dy1—O6—C14	−67.3 (7)
O6—Dy1—O1—C2	−166.8 (6)	O5—Dy1—O6—C14	0.8 (6)
O3—Dy1—O1—C2	−66.9 (6)	O1—Dy1—O6—C14	−122.4 (6)
O4—Dy1—O1—C2	−26.9 (7)	Dy1—O5—C12—C13	9.0 (11)
O8—Dy1—O1—C2	−149.8 (7)	Dy1—O5—C12—C11	−168.6 (5)
O7—Dy1—O1—C2	133.8 (7)	O5—C12—C13—C14	−2.2 (12)
O5—Dy1—O1—C2	83.7 (7)	C11—C12—C13—C14	175.4 (8)
O6—Dy1—O2—C4	161.1 (6)	Dy1—O6—C14—C13	3.7 (12)
O3—Dy1—O2—C4	44.6 (6)	Dy1—O6—C14—C15	−176.3 (5)
O4—Dy1—O2—C4	116.9 (6)	C12—C13—C14—O6	−4.2 (13)
O8—Dy1—O2—C4	−17.8 (8)	C12—C13—C14—C15	175.7 (8)
O7—Dy1—O2—C4	−95.3 (6)	O6—C14—C15—F9	−54.8 (11)
O5—Dy1—O2—C4	−159.4 (6)	C13—C14—C15—F9	125.2 (9)
O1—Dy1—O2—C4	−28.5 (6)	O6—C14—C15—F8	66.5 (10)
Dy1—O1—C2—C1	169.3 (5)	C13—C14—C15—F8	−113.5 (9)
C1—C2—C3—C4	169.1 (8)	O6—C14—C15—F7	−175.0 (8)
Dy1—O2—C4—C5	−158.1 (5)	C13—C14—C15—F7	5.0 (12)
C2—C3—C4—C5	−174.7 (7)	O2—Dy1—O7—C17	177.5 (6)
C3—C4—C5—F1	112.4 (10)	O6—Dy1—O7—C17	−41.1 (6)
O2—C4—C5—F2	55.4 (10)	O3—Dy1—O7—C17	79.6 (7)
C3—C4—C5—F2	−126.4 (9)	O4—Dy1—O7—C17	−95.9 (7)
O2—C4—C5—F3	173.3 (8)	O8—Dy1—O7—C17	29.3 (6)
O2—Dy1—O3—C7	42.0 (7)	O5—Dy1—O7—C17	−113.6 (6)
O6—Dy1—O3—C7	−100.4 (7)	O1—Dy1—O7—C17	110.9 (6)
O4—Dy1—O3—C7	−36.8 (7)	O2—Dy1—O8—C19	−119.0 (6)
O8—Dy1—O3—C7	−165.8 (7)	O6—Dy1—O8—C19	61.8 (5)
O7—Dy1—O3—C7	145.7 (6)	O3—Dy1—O8—C19	177.0 (6)
O5—Dy1—O3—C7	−9.3 (9)	O4—Dy1—O8—C19	116.3 (5)
O1—Dy1—O3—C7	114.5 (7)	O7—Dy1—O8—C19	−33.5 (5)
O2—Dy1—O4—C9	−60.3 (7)	O5—Dy1—O8—C19	11.7 (6)
O6—Dy1—O4—C9	147.6 (7)	O1—Dy1—O8—C19	−108.5 (6)
O3—Dy1—O4—C9	29.4 (7)	Dy1—O7—C17—C18	−18.4 (11)
O8—Dy1—O4—C9	93.9 (7)	Dy1—O7—C17—C16	163.5 (6)
O7—Dy1—O4—C9	−153.7 (6)	F10B—C16—C17—O7	−176.4 (14)
O5—Dy1—O4—C9	−137.0 (7)	F12B—C16—C17—O7	54.3 (17)
O1—Dy1—O4—C9	−10.8 (8)	F11B—C16—C17—O7	−54.0 (14)
Dy1—O3—C7—C6	−148.8 (6)	F10B—C16—C17—C18	5.4 (18)
C6—C7—C8—C9	179.6 (9)	F12B—C16—C17—C18	−123.9 (16)
Dy1—O4—C9—C10	166.1 (6)	F11B—C16—C17—C18	127.7 (12)
C7—C8—C9—C10	169.6 (8)	O7—C17—C18—C19	−5.8 (12)
O4—C9—C10—F6B	−20.3 (15)	C16—C17—C18—C19	172.2 (8)
C8—C9—C10—F6B	164.5 (13)	Dy1—O8—C19—C18	28.9 (10)
O4—C9—C10—F6	175.6 (11)	Dy1—O8—C19—C20	−155.5 (6)
C8—C9—C10—F6	0.5 (15)	C17—C18—C19—O8	−0.1 (12)
O4—C9—C10—F5	60.6 (12)	C17—C18—C19—C20	−175.5 (7)
C8—C9—C10—F5	−114.6 (12)	O8—C19—C20—F11	46.0 (14)
O4—C9—C10—F5B	−129.3 (15)	C18—C19—C20—F11	−138.1 (12)
O2—Dy1—O5—C12	−162.0 (7)	O8—C19—C20—F12	−79.5 (12)
O6—Dy1—O5—C12	−7.2 (7)	C18—C19—C20—F12	96.5 (12)
O3—Dy1—O5—C12	−108.4 (7)	O8—C19—C20—F10	163.7 (8)
O4—Dy1—O5—C12	−82.0 (7)	C18—C19—C20—F10	−20.3 (13)
O8—Dy1—O5—C12	41.9 (7)	C25—N1—C21—C22	1.3 (13)
O7—Dy1—O5—C12	88.7 (7)	N1—C21—C22—C23	−0.7 (14)
O1—Dy1—O5—C12	140.3 (6)	C21—C22—C23—C24	−1.4 (15)
O2—Dy1—O6—C14	40.7 (8)	C22—C23—C24—C25	3.0 (14)
O3—Dy1—O6—C14	149.1 (6)	C23—C24—C25—N1	−2.4 (14)
O4—Dy1—O6—C14	86.2 (7)	C21—N1—C25—C24	0.3 (14)
O8—Dy1—O6—C14	−139.9 (7)		

### Hydrogen-bond geometry (Å, °)

**Table:**

*D*—H···*A*	*D*—H	H···*A*	*D*···*A*	*D*—H···*A*
N1—H1···O5	0.86	2.10	2.947 (8)	167

## References

[bb1] Bruker (2000). *SMART* and *SAINT* Bruker AXS Inc., Madison, Wisconsin, USA.

[bb2] Chu, W. J. & Elgavish, G. A. (1995). *NMR Biomed.* **8**, 159–163. 10.1002/nbm.1940080404 8771090

[bb3] Farrugia, L. J. (1997). *J. Appl. Cryst.* **30**, 565.

[bb4] Iwamuro, M., Hasegawa, Y., Wada, Y. & Murakoshi, K. (1997). *Chem. Lett.* **10**, 1067–1068.

[bb5] Ma, B. Q., Zhang, D. S., Gao, S., Jin, T. Z. & Yan, C. H. (2000). *Angew* *Chem* *Int* *Ed* **39**, 3644–3646. 10.1002/1521-3773(20001016)39:20<3644::aid-anie3644>3.0.co;2-1 11091425

[bb6] Sheldrick, G. M. (2000). *SADABS* University of Göttingen, Germany.

[bb7] Sheldrick, G. M. (2008). *Acta Cryst.* A**64**, 112–122. 10.1107/S0108767307043930 18156677

[bb8] Tian, L., Ren, N., Zhang, J.-J., Sun, S.-J., Ye, H.-M., Bai, J.-H. & Wang, R.-F. (2009). *J* *Chem* *Eng* *Data* **54**, 69–74.

[bb9] Tsukube, H. & Shinoda, S. (2002). *Chem* *Rev* **102**, 2389–2404. 10.1021/cr010450p 12059273

